# 665 Global Burn Outcomes: Does Private or Government Ownership Status Matter?

**DOI:** 10.1093/jbcr/iraf019.294

**Published:** 2025-04-01

**Authors:** Daniel Najafali, Hilary Liu, Megan Najafali, Saeid Rezaei, José Arellano, Logan Galbraith, Mare Kaulakis, Francesco Egro

**Affiliations:** Carle Illinois College of Medicine; University of Pittsburgh Medical Center; Loyola University Chicago Stritch School of Medicine; University of Pittsburgh Medical Center; University of Pittsburgh Medical Center; Northeast Ohio Medical University; University of Pittsburgh School of Medicine; University of Pittsburgh Medical Center

## Abstract

**Introduction:**

Burn injuries are a major global concern with significant mortality and morbidity rates. Although there has been extensive research on the treatment and recovery process, the impact of institutional ownership on quality of care and burn outcomes is not understood on a global level.

**Methods:**

The data used in this study were obtained from the World Health Organization Global Burn Registry and accessed in September 2024. Hospitals were categorized based on ownership status as either private or governmental institutions. Key variables, including demographic information, burn characteristics, contributing factors, and burn outcomes, were analyzed. Descriptive analysis was employed to summarize these data, while multivariable logistic regression was conducted to assess associations with surgical intervention, physical impairment at discharge for survivors, and mortality.

**Results:**

Among the (N=9,274) cases analyzed, 7,055 (76%) were treated in a government facility, while 2,219 (24%) received their care in private facilities. In governmental hospitals, 38% were female, compared to 47% in private hospitals (P< 0.001). The median Baux Score was 43 for patients in governmental institutions, while for those in private settings, it was significantly higher at 50 (P< 0.001). The modified Baux score followed a similar trend, it was 44 (IQR: 21-72) in the government group compared to 52 (IQR: 28-80) in the private group (P< 0.001). Patients in governmental institutions had a median TBSA of 15%, whereas those treated in private institutions exhibited a higher median TBSA of 20% (P=0.033). Household and occupational accidents were significantly more frequent among patients treated in private institutions compared to those in government settings (P< 0.001). Patients in government hospitals had a median stay of 10 days (IQR: 5 to 18 days), while those in private hospitals had 9 days (IQR: 4 to 17) (P< 0.001). Mortality and impairment were significantly higher in government hospitals compared to private hospitals (P< 0.001). Private hospitals demonstrated significantly higher odds of undergoing surgery after burn injury (OR 2.61, 95%CI 2.32-2.95) and lower odds of mortality (OR 0.40, 95% CI 0.32-0.49).

**Conclusions:**

Ownership status in healthcare delivery plays a critical role in shaping burn patient outcomes on a global scale. Government hospitals, handling most cases, report higher mortality and impairment rates, whereas private hospitals show better outcomes and shorter hospital stays. These findings suggest that improving public healthcare systems, particularly in low-resource settings, could reduce disparities in outcomes.

**Applicability of Research to Practice:**

Assessing the impact of ownership status on burn centers and improving the infrastructure of public healthcare systems could greatly improve burn patient outcomes.

**Funding for the Study:**

N/A

